# Supplementary material for: Diagnosis of DSD in Children—Development of New Tools for a Structured Diagnostic and Information Management Program within the Empower-DSD Study
Source: J Clin Med. 2022 Jul 3;11(13):3859. doi: 10.3390/jcm11133859 (PMC9267843; doi:10.3390/jcm11133859)
Supplement: Supplementary file 1 [file jcm-11-03859-s001.zip › jcm-1772655-supplementary.pdf]

Table S1: existing information resources (last updated April 2022)

a) Included to be passed to the family in "my record"

Web page	Brochure	Content
https://www.ags-initiative.de/	„AGS – Die unschlimmste Erkrankung, die man sich vorstellen kann" Publisher: AGS Eltern- und Patienteninitiative e.V.: 2017	Information on diagnosis and treatment of CAH
https://im-ev.de/	„Wenn das Erscheinungsbild Ihres Kindes in kein Geschlecht zu passen scheint…" Publisher: Intergeschlechtliche Menschen e.V. Bundesverband, 2020	Information for parents of a newborn with DSD
https://www.dsd-life.eu/home/index.html	„Wenn Ihr Kind bei der Geburt nicht wie ein typisches Mädchen oder ein typischer Junge aussieht. Die ersten Tage" Publisher: dsd families translated by dsd-LIFE, 2015	Information for parents of a newborn with DSD
https://www.ags-initiative.de/	„Lisa, Paul und das AGS" Publisher: AGS Eltern- und Patienteninitiative e.V.	Short story to explain the pathophysiology of CAH to children.

b) Not included

Web page	Brochure	Not included because
https://www.glandula-online.de/	„Adrenogenitales Syndrom mit 21-Hydroxylase-Defekt (AGS)" Publisher: Netzwerk Hypophysen- und Nebennierenerkrankungen e. V	Language not inclusive and medical words not explained in everyday language.
https://www.bmfsfj.de/resource/blob/94014/525976078568376b94a029fff875aea8/ihr-intergeschlechtliches-kind-data.pdf	„Weiblich? Männlich? Ihr intergeschlechtliches Kind." Publisher: QueerLeben, Berlin	Contact details of peer support. No medical information, no date of publication
https://im-nds-ev.de/wp-content/uploads/2021/06/Broschuere_IMLVNDSeV_2021_web.pdf	„Intergeschlechtlich in Niedersachsen" Publisher: Intergeschlechtliche Menschen Landesverband Niedersachsen e.V.	Not applicable to all federal states in Germany
https://www.dsdfamilies.org/	• The story of sex development<br>• Nurseries and your son<br>• Top tips for dilation<br>• Top tips for talking about differences of sex development	English language
https://www.accordalliance.org/	• Lend a Helping Hand: a Resource Guide for DSD Care<br>• Handbook for parents	English language

c) Materials directed towards health care professionals

Web page	Brochure	Content
https://im-ev.de/	Was ist es denn? Intergeschlechtlichkeit / DSD Ein Ratgeber für Hebammen / Geburtshelfer*innen Publisher: Intergeschlechtliche Menschen e.V. Bundesverband, 2021	Information for midwives/obstetricians of a newborn with DSD
